# Consumer Perception and Sensory Drivers of Liking of Fortified Oat Milks

**DOI:** 10.3390/foods12224097

**Published:** 2023-11-11

**Authors:** Christy Alsado, Laura Lopez-Aldana, Lingyun Chen, Wendy Wismer

**Affiliations:** Department of Agricultural, Food and Nutritional Science, University of Alberta, Edmonton, AB T6G 2P5, Canada; christym@ualberta.ca (C.A.); llopez1@ualberta.ca (L.L.-A.); lingyun1@ualberta.ca (L.C.)

**Keywords:** plant-based milk, oats, β-glucan, protein, product development, consumer acceptance

## Abstract

Oat milk was fortified with β-glucan at a level that attains health benefits and protein at a level equivalent to that of cow’s milk. This study aimed to identify consumer perceptions and evaluate the sensory attributes of fortified plain and chocolate oat milks. Oat milk consumers (*n* = 106) evaluated four samples: C (Control), 0Pro (6.25 g/L β-glucan), LPro (6.25 g/L β-glucan and 15.23 g/L oat protein), and HPro (6.25 g/L β-glucan and 30.45 g/L oat protein); and they completed free-word association (FWA), liking ratings, just-about-right (JAR), check-all-that-apply (CATA), and conjoint analysis (CA). Oat milk was associated with sensory descriptors, environmental sustainability, and health benefits. C and 0Pro products were liked significantly more than LPro and HPro. C and 0Pro oat flavors and thicknesses were rated ”just about right” by majority of the participants, while LPro and HPro were rated “too much”. Positive CATA attributes were “smooth”, “fresh”, and “oat-like” while negative attributes were “rancid”, “sandy”, and “grainy”. The CA results showed consumer interest in oat milk fortified with oat protein, containing β-glucan at a level recommended for health benefits, and with protein levels higher than cow’s milk. Based on the results, β-glucan-fortified oat milk is acceptable while oat protein fortification requires reformulation or substitution with another source.

## 1. Introduction

Among plant-based products, milk alternatives comprise the largest category, with an 11.4% Compound Annual Growth Rate (CAGR) from 2017 to 2022. This category is projected to expand with a 7.0% CAGR increase in 2022–2027 [1]. The growing trend of plant-based milk consumption in recent years derived primarily from consumer demand for products with health-promoting advantages influenced by functional ingredients and nutritional composition [2]. The increased consumption of plant-based milks was also the result of increasing milk protein allergy and lactose intolerance, as well as awareness of environmental and ethical concerns of bovine milk production [3,4]. 

Plant-based milks are typically sourced from cereals, legumes, nuts, seeds, and pseudo-cereals [5]. Oat milk was the second largest plant-based milk category in 2020 and continued with a 131.9% CAGR. The market share is projected to grow through to 2027 with increased product launches in Europe, Australia, New Zealand, and North America [1,6]. 

Oats are an excellent source of proteins; dietary fibers, mainly β-glucan; lipid components containing antioxidants; and phytochemicals [7]. It contains 15–20% proteins with a good amino acid balance [8]. However, oat milk contains 3 g of protein per 240 mL serving as compared to the 7.69 g of protein per serving of cow’s milk. Cow’s milk provides around 60% of the protein requirement of a young child aged 1–3 years old, while oat milk provides around 23% of protein of the same age group [9]. 

Another important functional property of oats is the ability to lower blood total cholesterol level and moderate glycemic response, attributed to its highly viscous β-glucan component [10]. Oats generally contain about 3% to 7% β-glucan depending on the varieties [11]. Canadian statutory accepted health claims state that a 0.75 g/serving or 3 g/day dosage of β-glucan from oats would significantly reduce the risk associated with coronary heart disease [12,13], reduce blood glucose, and improve satiety [14]. However, the European Food Safety Authority Panel concluded that obtaining a consistent reduction in postprandial glycemic responses requires a dosage of 4 g of oat β-glucan per 30 g of available carbohydrate [15]. Moreover, a clinical study presented the need to enrich β-glucan content in beverages to a 5 g dosage to provide an overall positive metabolic effect and cause a significant reduction in postprandial glucose, insulin, and total cholesterol concentration [16]. While current brand innovations center oat milk fortification on calcium and vitamins A and D, market reports reveal consumer interest in products that support immune health through fibers such as β-glucan and protein, creating an opportunity for the fortification of the plant-based milks derived from oats, which are sources of these nutritive compounds [17,18].

However, nutrient fortification in food formulations may cause significant changes in composition and physicochemical properties, which subsequently impact the sensory properties [19] such as flavor and mouthfeel, in turn affecting consumers’ overall liking of the product [20]. Ensuring that the sensory attributes of developed plant-based milks are acceptable to consumers is essential to increase the chances for success in the market [21]. Most marketed beverages are fortified with antioxidants, vitamins, collagen, and protein [22] while limited studies have been conducted on β-glucan fortified plant-based milks. An oat variety with the highest β-glucan content showed a better oat milk flavor and appearance; however, further studies are still required [23]. 

With this, the aim of this study was to identify the oat milk consumer perception of this product, develop and evaluate the sensory attributes of plain and chocolate oat milks fortified with β-glucan and protein, and identify extrinsic attributes that influence consumer acceptance of a hypothetical fortified oat milk. 

## 2. Materials and Methods

**Figure 1:**
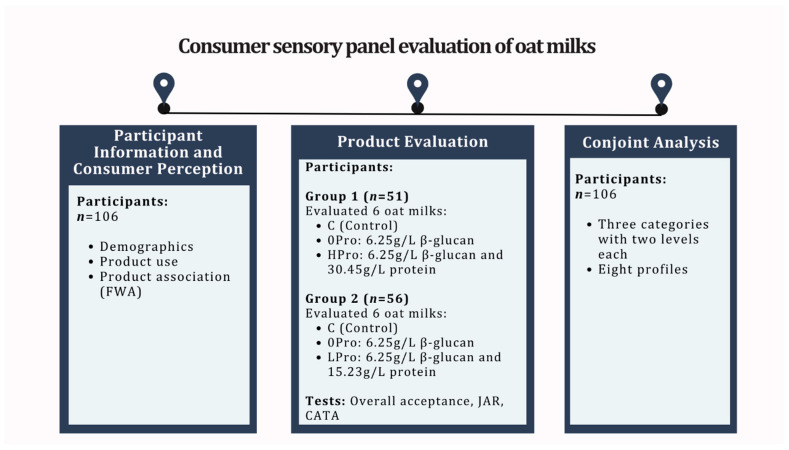
Overview of the study design.

The study was approved by the University of Alberta Research Ethics Committee (Pro000118690) on 7 September 2022. The overall study design had two parts: the preliminary screening that identified the workable levels of β-glucan and protein added to oat milk; and a consumer sensory panel evaluation that included demographics, product use information, product tasting, and conjoint analysis (Figure 1). All testing was conducted in the Sensory and Consumer Science Laboratory at the University of Alberta using standard sensory booths (ISO 8589) [24], and the data were collected using Compusense Cloud (Guelph, ON, Canada).

### 2.1. Preliminary Screening and Formulation of β-Glucan and Protein Levels

#### 2.1.1. Sample Preparation

Plain and chocolate-flavored oat milks in 946 mL shelf-stable packs obtained from Earth’s Own Food Company, Inc. (Burnaby, BC, Canada), were purchased from a local supermarket. The β-glucan (34% concentration) and protein (55% concentration) used were commercial oat products purchased from Lantmannen Functional Foods AB (Stockholm, Sweden). For each flavor of oat milk, nine (9) samples were prepared following a 2^3^ factorial design with three fortification levels for β-glucan (5.88 g/L, 6.25 g/L, and 6.62 g/L) and protein (29.09 g/L, 30.45 g/L, and 31.82 g/L). The β-glucan levels were based on the daily dosage necessary to attain health benefits [15,16], while the protein levels were selected to match the protein content in cow’s milk. 

β-glucan was gradually added to water with constant stirring with a digital stirrer (Caframo Lab Solutions; Wiarton, ON, Canada) until all particles dissolved. The solution was heated to 70 °C for ten minutes [25] and cooled to 35 °C before oat milk was added. For samples containing β-glucan and protein, a portion of oat milk was blended with protein and then mixed into the oat milk with β-glucan. The samples were stored at refrigeration temperature (4–7 °C) until the testing day. Three batches with three formulations were prepared for every screening session.

#### 2.1.2. Screening Panel

A screening panel composed of seven experienced assessors from the University of Alberta, with experience in food sensory evaluations and 16 h of experience in evaluating oat milk, was formed to identify testable levels of β-glucan and protein added to oat milk. Three samples of each oat milk flavor were presented to the panel for every screening session. An intensity scale was used to evaluate the color, taste, mouthfeel, and overall sensory attributes. The 9-point hedonic scale was used to ensure that the samples fortified with levels recommended by the panel had a high acceptance rating suitable for advancement to the consumer sensory panel evaluation. The panel also modified the CATA list developed by Oliveria et al. [26] for applicability to oat milk.

### 2.2. Consumer Sensory Panel Evaluation of Oat Milks

#### 2.2.1. Consumer Panelist Recruitment

Participants were recruited from the University of Alberta community through online list servers and flyers. The criteria for panelist inclusion included liking and regular consumption of oat milk (at least once a month). Participants confirmed their eligibility and provided written informed consent before the evaluation. A total of 106 participants participated in the study. Each participant received a small gift card after the sensory session. 

#### 2.2.2. Selected Formulations and Sample Presentation

Three samples were presented to the consumers: C (Control), 0Pro (fortified 6.25 g/L with β-glucan), and HPro (fortified with 6.25 g/L β-glucan and 30.45 g/L protein). Samples of 70 mL portions were dispensed in 100 mL sample cups with a lid and labeled with 3-digit codes. The samples were presented simultaneously with the control placed first in the series followed by two other samples in a Balanced Design (BD). 

At the mid-point of the evaluation (*n* = 51), despite preliminary screening, results for the overall liking of HPro showed a very low acceptability. Consequently, the HPro sample was replaced with LPro (fortified with 6.25 g/L β-glucan and 15.23 g/L protein) for the subsequent evaluations. This created two groups of evaluations with 51 participants having evaluated samples C, 0Pro, and HPro; and 56 participants having evaluated samples C, 0Pro, and LPro. The same treatments were used for chocolate oat milks labeled as CC, C0Pro, CLPro, and CHPro, respectively (Table 1). 

#### 2.2.3. Sensory Test Methods and Questionnaire

Participants were initially asked to complete demographic questions, followed by Free Word Association (FWA), which invited participants to write down four words (images, associations, thoughts, or feelings) that come to mind when thinking of oat milks. The participants then completed the sensory evaluation of the oat milks. Conjoint analysis was completed after all products had been evaluated. 

The sensory questionnaire included JAR-acceptance [27] and CATA [26,28] evaluations. In the JAR evaluation, oat flavor and thickness were assessed using 5-point scales with the left endpoint labeled as “Too Little”, middle point as “Just About Right”, and right endpoint as “Too Much” [27]. In the overall acceptability, participants rated liking using a 9-point hedonic scale (1 = dislike extremely, 9 like very much). Participants also selected applicable CATA descriptors from the 24 terms modified by the screening panel: *oat-like*, *salty*, *sweet*, *sour*, *bitter*, *strong*, *mild*, *strange*, *soft*, *grainy*, *smooth*, *sticky*, *greasy*, *nutty*, *roasted*, *musty*, *fresh*, *healthy*, *natural*, *artificial*, *easy*, *chalky*, *sandy*, *and rancid*.

### 2.3. Conjoint Analysis

Participants rated six of eight hypothetical product profiles generated from three categories with two levels of each in an orthogonal design (Table 2). Ratings were made on a 9-point hedonic scale (1 = dislike extremely, 9 like very much), and the optimized design had a reduced number of profiles to minimize panelist fatigue during evaluation [29]. The profiles were generated using XLSTAT software version 2022.4.1 (Lumivero, Denver, CO, USA). 

### 2.4. Data Analyses

Demographic and product use data were analyzed using descriptive statistics. FWA results were analyzed using methods described by Moss et al. [30]. The frequency of mention was determined, and elicited words were grouped into categories and sub-categories identified by the authors. The sub-categories were reported as percentages mentioned by at least 5% of participants. Analysis of variance (ANOVA) was used to establish differences in the overall liking means, followed by Tukey’s multiple-comparison test to establish differences among samples, when appropriate. JAR responses were analyzed using descriptive statistics to present frequencies across the scale and identify where the samples were centered. JAR results combined with liking scores were also analyzed using penalty analysis [27]. In CATA, the frequency of each term was determined using the number of participants that used the term to describe each sample. Multiple Factor Analysis (MFA) was used to establish relationships from the CATA frequency table with attributes as variables and overall liking as the supplementary variable [31]. Each observation was assigned a weight that is a function of the frequency of the corresponding category [32] with C (*n* = 106), 0Pro (*n* = 106), LPro (*n* = 55), and HPro (*n* = 51). Bootstrap analyses were conducted to verify the significance of an attribute to a dimension, when appropriate. 

For conjoint analysis, mixed-model ANOVA was used to establish differences among the profiles. The model considers the following responses: consumer rating data as measured response and main effects; interaction of conjoint factors as fixed effects; consumer effects and its interaction with conjoint factors as random effects; and error term [29]. All other statistical analyses were conducted using XLSTAT software version 2022.4.1 (Lumivero, Denver, CO, USA) in Microsoft Excel^TM^ at a significance value of *p* ≤ 0.05. 

## 3. Results and Discussion

### 3.1. Demographics 

Among the 106 study participants, the majority were females (70%) and between 18 to 34 years old (95.29%). Most of the participants were post-secondary attendees or graduates (95.28%) and consumed oat milk at least once a month (100%), with the original and unsweetened original as the most consumed forms, and the Silk and Earth’s Own brands as the most used; the main uses of oat milk were as dairy milk alternatives and as coffee creamer (Table 3). 

According to a 2022 market report, more females than males were consuming plant-based milk with a higher inclination to follow vegan and vegetarian dietary preferences and special diets (weight loss). Most females were also the primary shoppers influencing the selection of products consumed in a household. Plant-based consumers were typically 18–35 years old. This age group is more receptive to plant-based milk alternatives compared to consumers aged 55 years and older [1]. A faster rate of adoption was observed among consumers who attended college or post-secondary education as compared to high school graduates [33]. The main uses of plant-based milks are as coffee creamer and an ingredient in cooked dishes and baked goods [22]. 

The close alignment of the demographic characteristics and oat milk consumption habits of the participants in this study with current oat milk consumer profiles described in the general population confirms the generalizability of our consumer and sensory perceptions to those of current oat milk consumers. 

### 3.2. Free Word Analysis

Participants (*n* = 106) generated a total of 424 words with responses in the form of single words and short phrases, which were grouped into 12 categories (Table 4). The results showed that the category of “sensory descriptors” was most mentioned, and taste associations were mostly positive words and some negative words such as “off-texture”, “grainy”, and “watery”. This reveals that consumers identified sensory attributes as the dominant characteristics of plant-based milks [30]. 

The second most mentioned category was “environment”, signifying consumer interest in plant-based products which are linked to attaining a sustainable food supply with reduced climate impacts and environmental footprints, addressing the new-generation consumer demand for sustainable products [34,35]. A food and health survey confirmed an increase from 2019 to 2021 in the number of consumers who placed importance on environmental sustainability [36]. The intensive promotion of sustainable benefits through media and product marketing has increased awareness to general consumers. About 39% agreed on environmental benefits, while 33% believed that plant-based milks are healthier [1]. 

The “health” category identified the health benefits of oat milks and intersected with the “nutrition” category, which mentioned oat milk as being “nutritious”, “low calorie”, and “low fat”. The “uses” category also mentioned a healthier alternative to milk. Oats are ideal milk alternatives addressing the problem of milk allergies, lactose intolerance, and celiac diseases [4,19,23]. The remaining categories with less than 5% of citations mentioned emotions (comfort, warm, enjoyable, satisfying); opinions (cost, newness of product); flavors (variants of oat milks and oat products); brands or locations (coffee shop brands, grocery locations); milk (terms relating to milk); and others.

Asking respondents to freely associate what ideas come to mind provides spontaneous and unbiased thoughts [37], which identifies essential product characteristics liked or disliked by consumers, food choice motivations, purchase intentions, and possible improvements for the product [38]. The positive associations in the FWA results indicated that the oat milk consumers were familiar with the product and the source and considered it trustworthy. This implies that the product had a positive impact on consumer choice even when some product characteristics were disliked [39,40].

### 3.3. Overall Liking of Oat Milks, Just-about-Right, and Penalty Analysis

The mean scores of overall liking of plain oat milks ranged from dislike slightly to like slightly (Table 5). C and 0Pro showed significantly higher liking scores than LPro and HPro. Similarly, the overall liking means of chocolate samples ranged from dislike slightly to like moderately. CC had the highest overall liking score, followed by C0Pro, whereas CLPro and CHPro were least liked. 

The oat flavor and thickness of C were rated as JAR by 56.60% and 48.11% of participants, similar to 0Pro, which was rated as JAR by 55.66% and 64.15% of participants. However, LPro and HPro were rated as having “too much” of both attributes. The results of chocolate oat milk evaluations followed the same trend. 

**Figure 2:**
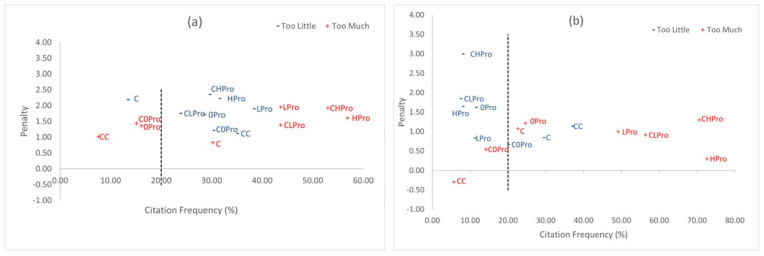
Penalty analysis plots for plain and chocolate oat milks for the attributes: (**a**) oat flavor; (**b**) thickness; sample identity: C (Control), 0Pro (fortified 6.2 5g/L with β-glucan), LPro (fortified with 6.25 g/L β-glucan and 15.23 g/L protein), and HPro (fortified with 6.25 g/L β-glucan and 30.45 g/L protein); same treatments were used for chocolate oat milks labeled as CC, C0Pro, CLPro, and CHPro, respectively.

The penalty analyses of JAR evaluations (Figure 2a,b) determine if the attributes are perceived to be optimal or in which direction the product can be improved to increase liking [27,41]. A penalty score of >1.0 and an occurrence of >20% was considered detrimental to the overall liking in this study. The attributes plotted at the upper right quadrant indicate a large penalty given by a large number of consumers and can be the focus for product reformulation [42].

The addition of 6.25 g/L β-glucan significantly increased overall liking and the thickness of the samples. The addition of plant polysaccharides in beverages thickens the liquid phases and increases viscosity which increases creaminess and improves mouthfeel, mimicking dairy milk [43,44]. Replicating these dairy milk properties is important to ensure consumer adoption as plant-based milks have lower acceptability due to their gritty mouthfeel and lower viscosity [22,45]. However, oat milk fortification with both β-glucan and protein significantly decreased the overall liking and was characterized by participants as having “too much” oat flavor and thickness. 

The majority of participants penalized the products having “too much” oat flavor, which was observed in samples fortified with protein (LPro and HPro). Oat protein has a high fat-binding capacity due to its lower bulk density; its addition entraps oil, and it is responsible for retaining flavor and texture during processing [46]. Products having “too little” oat flavor were also penalized by a minority of participants. These were perceived in samples fortified with β-glucan (0Pro). The addition of β-glucan increases the hydrocolloid content causing a decrease in perceived flavor in beverages [20,47]. 

The majority of participants also penalized the products with “too much” thickness. Similar to oat flavor, the number of participants who rated “too much” thickness increased upon increasing protein fortification. Oat protein has a low solubility, which limits its application in liquid products [8]. The oat protein used in this study has a lower solubility than the water-soluble β-glucan; thus, a higher protein content generally contributes to more solids in the formulation. This also increases the viscosity, which negatively influences the mouthfeel [20,48] and further led to a decrease in the acceptance of the samples in this study. 

While the study products with “too much” thickness were strongly penalized, the largest penalty was given to products with “too little” thickness, with a 3.0 mean drop on the hedonic scale. This agrees with the findings of Vasquez-Orejarena et al. [20], where mouthfeel and thickness had the most relevant influence on the product as compared to other sensory attributes. The results of JAR in this study indicate that oat milks fortified with both β-glucan and protein should be reformulated to improve acceptability. 

### 3.4. Check-All-That-Apply and Multi-Factor Analysis

Participants checked a range of 1 to 11 CATA attributes per sample. For both plain and chocolate, the most cited terms were “oat-like” and “smooth”, while the least cited were “greasy”, “sour”, and “rancid”. 

**Figure 3:**
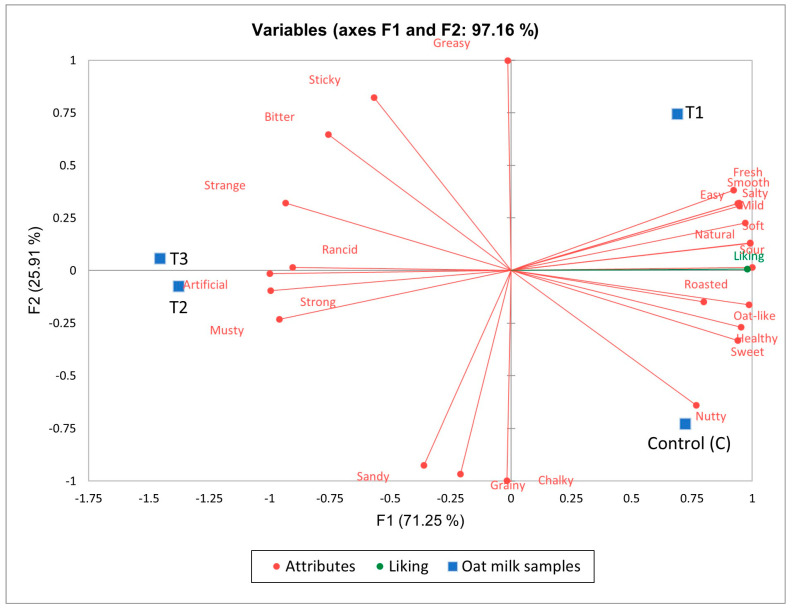
Variable correlation circle of multifactor analysis for plain oat milks.

For plain oat milk, the results of MFA showed that 97.16% of the experimental data variance was associated with the first three dimensions, with values indicating the significance of each dimension as influenced by the components [49]. The first dimension accounting for 71.25% variance (Figure 3) showed a positive correlation with the attributes sour, soft, oat-like, natural, mild, and salty, which indicated drivers of liking; and a negative correlation with the attributes strong, musty, strange, and rancid, which influenced consumers’ disliking of the oat milks. While the two least cited terms sour and salty showed a positive correlation, bootstrap values were 0.015 and 0.986, respectively, which were less than the critical value of 3.00 [49] and, thus, had no significant influence on the first dimension. 

The second dimension represented by a 25.91% variability correlated with the positive attributes greasy and sticky against the negative attributes grainy, sandy, and chalky. The positive terms relate to thickness, fattiness, and creaminess, which are dominant attributes in fluids and dairy foods [50], while the negative terms relate to mouthfeel. The third dimension of a 2.84% variability provides little information. 

The samples were dispersed in the four quadrants of the variable correlation circle, with C and 0Pro correlated with the positive attributes nutty and smooth, respectively. Oats are characterized by desirable sensory properties such as nutty and cereal-like odor [44,51], and the fortification of β-glucan showed a perceptible smoothness of the product. However, LPro and HPro were correlated with the negative attributes rancid, artificial, and strong. The fortification of protein strongly affects the flavor of the product, as lipids residues in the protein samples could possibly lead to hydrolytic and oxidative deterioration that further develops into a bitter taste and rancid flavor [34,44].

**Figure 4:**
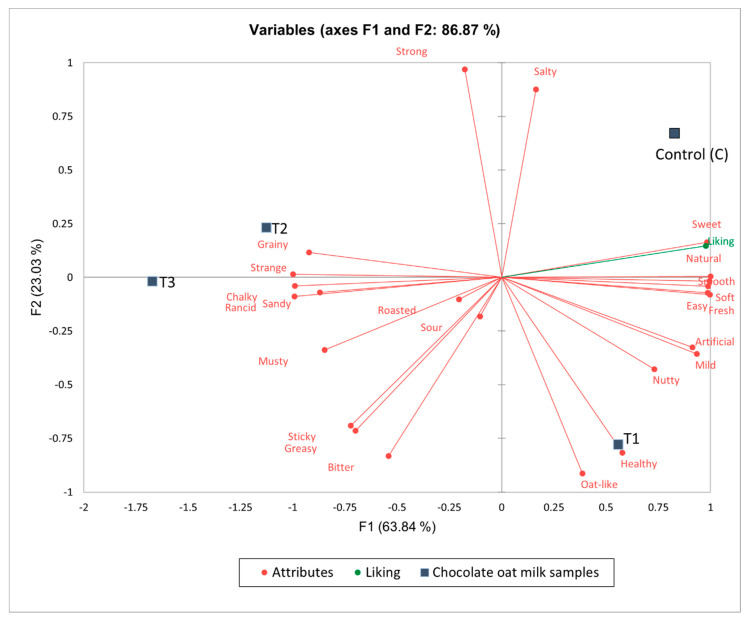
Variable correlation circle of multifactor analysis for chocolate oat milks.

The MFA results of chocolate oat milk indicate an 86.87% variance from three dimensions. Based on factor loadings, the first dimension accounting for a 63.84% variance (Figure 4) showed a positive correlation with the attributes smooth, natural, fresh, soft, easy, and sweet; and a negative correlation with the attributes strange, chalky, rancid, and sandy. 

The second dimension accounting for a 23.03% variance showed a positive correlation with strong and salty against the attributes oat-like and bitter. Moreover, the third dimension with a 13.13% variance showed a positive correlation with sour and roasted against artificial, grainy, and sandy. Most of the attributes were in the bisector and were contrasted according to intensity, flavor, and mouthfeel. The last two dimensions did not provide much information as compared to the first dimension.

The chocolate samples were also dispersed in the four quadrants of the variable correlation circle. CC was positively correlated with sweet; C0Pro was positively correlated with oat-like and healthy. Moss et al. [30] found that chocolate variants of plant-based milk were liked more than the unsweetened counterparts as the flavor masks some of the negative sensory attributes such as off and the beany flavor. The addition of β-glucan may have reduced the sweetness but intensified the oat flavor of our samples. However, CLPro and CHPro were correlated to the attributes grainy, strange, chalky, rancid, and sandy, indicating that the addition of protein has a consistent negative effect on mouthfeel even with the chocolate flavoring.

### 3.5. Conjoint Analysis

**Figure 5:**
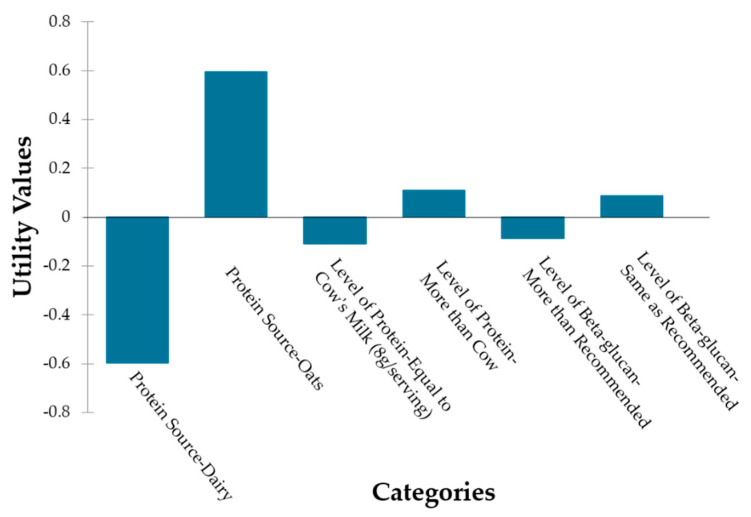
Conjoint analysis of average utilities of the oat milk attributes.

Participants’ responses to hypothetical oat milk concepts (Figure 5) showed that the protein source category had the highest relative importance of 44.67%, followed by the level of β-glucan and level of protein with a relative importance of 28.91% and 26.35%, respectively. 

The protein source was the most influential variable, with oat protein having a positive utility value of (β = 0.60) while the use of dairy protein had a negative utility value. The trend of choosing plant-based alternatives is influenced by consumer awareness and increased demand for sustainable products. Oat protein is also a good option for consumers as it contains a higher protein content with a balanced amino acid profile as compared to other cereals [8]. 

While the relative importance of the level of β-glucan and the level of protein did not largely differ, adding more than the recommended level of β-glucan had a negative utility value (β = −0.087), and adding protein content more that that of cow’s milk (>8 g protein/serving) had a positive utility value (β = 0.108). 

This might be influenced by a lack of interest in β-glucan levels greater than recommended levels or a lack of familiarity with β-glucan among the study participants. Most oat products that qualify for β-glucan claims are labeled with the fiber content [13], which makes consumers more familiar with the term “fiber” as it is frequently promoted as an important dietary component. Consumers also have more positive perceptions of products with ingredients listed in common names rather than scientific names [52], which plays a significant role in consumer attitude and purchase intentions [53]. 

Moreover, protein fortification is a challenge for plant-based milks to be at par with the protein content of dairy milk [54]; however, consumer awareness of the functional ingredient’s health benefits increases the product attractiveness [53]. 

This study identified the consumer perception of oat milk and developed and evaluated sensory attributes of fortified oat milk. The study participants’ demographics aligned with those of oat milk users profiled in recent market studies [33]. In FWA, the most mentioned category was “sensory descriptors”, indicating that oat milk’s sensory attributes are most important to consumers. The β-glucan fortification of oat milk also improved the liking and made it to be perceived as just-about-right by majority of the participants; however, oat milk fortified with β-glucan and oat protein was perceived to have “too much” oat flavor and thickness, which significantly decreased the overall liking, even with the chocolate flavoring. 

Oat milk fortification with β-glucan and oat protein provides a potential product comparable to the nutritional profile and sensory attributes of cow’s milk. The sensory attributes and drivers of liking of plain and chocolate oat milks fortified with β-glucan and protein were determined. This nutritious product may be of interest to both non-oat milk consumers and other individuals exploring plant-based milk options. The sensory profiles of other fortified plant-based milk and other derived products can be identified using the sensory methods and terms established in this study. 

Future studies could take advantage of an oat protein isolate (OPI) with a higher protein content than that used here, which would reduce the amount of fortificants in the formulation. This will potentially improve mouthfeel and reduce the rancid flavor and chalkiness that were identified as negative attributes in CATA. The use of other plant-sourced protein isolates such as pea and faba proteins, which have been recently used in beverage formulation, to improve plant-based milks’ functional and sensory properties is also a potential option, as the conjoint analysis results emphasized the importance of the protein source and level. Home-use studies conducted over multiple days permit investigation of how consumption in natural conditions and extended product exposure can influence acceptability and consumer behavior.

## 4. Conclusions

FWA oat milk concepts relevant to oat milk consumers were associated with sensory characteristics, environment, health, and nutrition. Among the study samples, products fortified with β-glucan (6.25 g/L) were acceptable while products fortified with both β-glucan (6.25 g/L) and oat protein (15.23 g/L or 30.45 g/L) were disliked by participants. The addition of β-glucan positively influenced the just-about-right oat flavor and thickness, while protein addition resulted in an excessive perception of the attributes. CATA’s positive drivers of liking were oat-like, natural, smooth, and fresh, contrary to the attributes rancid, artificial, strong, musty, strange, chalky, and sandy, which influenced consumer disliking. The addition of β-glucan and oat protein in the formulation had negative sensory consequences. However, the conjoint analysis revealed that the addition of oat protein is an attractive concept, thus the need to improve the quality of fortified products or the use of protein alternatives. 

## Figures and Tables

**Table 1:** Formulation of plain and chocolate oat milks fortified with β-glucan and protein per 1000 g of product.

Ingredient	0Pro/C0Pro (g)	LPro/CLPro (g)	HPro/CHPro (g)
Oat milk	868.75	853.52	838.30
β-glucan	6.25	6.25	6.25
Protein	0.00	15.23	30.45
Water	125.00	125.00	125.00

**Table 2:** Attribute categories and levels used in conjoint analysis to generate hypothetical oat milk profiles.

Categories	Levels	
Protein Source	Level of Protein	Level of β glucan
Dairy	Equal to cow’s milk (8 g/serving)	More than recommended (>3 g/day)
Oats	More than cow’s milk (>8 g/serving)	Same as recommended (3 g/day)

**Table 3:** Demographics of oat milk consumer participants evaluating the study on oat milks (*n* = 106).

	Number of Participants (%)
**Sex**	
Female	74 (69.81)
Male	30 (28.30)
Other	2 (1.89)
**Age range**	
18–24 years	81 (76.42)
25–34 years	20 (18.87)
35 and older	5 (4.72)
**Level of education**	
Some or completed high school	5 (4.72)
Some or completed College, Technical, University	66 (62.26)
Some or completed Post-Graduate Degree	35 (33.02)
**Frequency of oat milk consumption**	
Almost daily	26 (24.53)
Once per week	21 (19.81)
2–4 times per week	21 (19.81)
1–3 times a month	33 (31.13)
Never ^1^	5 (4.72)
**Forms of oat milk consumed ^1,2^**	
Unsweetened	56 (52.83)
Original	68 (64.15)
Unsweetened vanilla	24 (22.64)
Vanilla	36 (33.96)
Chocolate	28 (26.42)
Oat barista edition	23 (21.70)
Oat nog	9 (8.49)
Organic oat milk	10 (9.43)
Cinnamon	3 (2.83)
Strawberry and banana	2 (1.89)
**Oat milk brands used ^1,2^**	
Oatly	21 (19.81)
Earth’s own	63 (59.43)
Planet Oat	10 (9.43)
Silk	70 (66.04)
Califa	8 (7.55)
Chobani	9 (8.49)
Simple beverage	6(5.66)
**Oat milk uses ^1,2^**	
Alternative for dairy milk	82 (77.36)
As coffee creamer	60 (56.60)
Base for smoothie and shakes	52 (49.06)
As an ingredient for dishes or baked goods	34 (32.08)
Use for overnight oats or breakfast cereals	53 (50)

^1^ Participants interpreted the question as the use of oat milk as a stand-alone beverage but confirmed other uses of oat milk. ^2^ Total percentage exceeds 100 as some participants reported multiple forms, brands, and uses of oat milks.

**Table 4:** Categorized words mentioned in free word analysis to describe oat milk.

Category	Examples of Most Relevant Terms	* Percentage of Mention (*n* = 424)
Sensory Descriptors		40
	Taste (oaty, sweet, neutral, nutty)	
	Texture (creamy, smooth, thick, grainy)	39
	Overall perception (tasty, delicious, yummy, good taste)	35
	physical characteristics	18
Environment		12
	Environmental impact	47
	Nature	26
	Vegan	27
Health		10
	Healthy, beneficial	39
Uses		9
	Alternative	
	Breakfast	
	Baking	
Beverages		8
	Coffee	58
	Latte	27
Nutrition		7
	Lactose-free	55
	Low nutrients/calorie	17

* Each participant (*n* = 106) could provide up to four words.

**Table 5:** Mean scores (±standard deviation) for overall liking of the fortified oat milks (*n* = 106).

Samples	Overall Liking ^1,2^
**Plain oat milk**	
Control (C)	6.3 ± 1.49 a
0Pro	6.3 ± 1.63 a
LPro	4.4 ± 1.71 b
HPro	3.6 ± 1.68 c
**Chocolate oat milk**	
Control (CC)	7.3 ± 1.41 a
C0Pro	6.6 ± 1.63 b
CLPro	5.2 ± 1.84 c
CHPro	3.8 ± 1.90 d

^1^ 9-point hedonic scale (1 = dislike extremely, 9 = like extremely). ^2^ Means of samples having the same letter within the same oat milk flavor (plain or chocolate) are not significantly different (*p* < 0.05).

## Data Availability

The data presented in the study are available upon request from the corresponding author. The data are not publicly available for ethical reasons.

## References

[1] Rasch C. (2022). Dairy & Egg Alternatives: Plant-Based & Animal-Free Products.

[2] Nawaz M.A., Tan M., Øiseth S., Buckow R. (2020). An Emerging Segment of Functional Legume-Based Beverages: A Review. Food Rev. Int..

[3] Zhang Y.Y., Hughes J., Grafenauer S. (2020). Got Mylk?. The Emerging Role of Australian Plant-Based Milk Alternatives as A Cow’s Milk Substitute. Nutrients.

[4] Fructuoso I., Romão B., Han H., Raposo A., Ariza-Montes A., Araya-Castillo L., Zandonadi R.P. (2021). An Overview on Nutritional Aspects of Plant-Based Beverages Used as Substitutes for Cow’s Milk. Nutrients.

[5] Sethi S., Tyagi S.K., Anurag R.K. (2016). Plant-Based Milk Alternatives an Emerging Segment of Functional Beverages: A Review. J. Food Sci. Technol..

[6] Walji A. (2023). A Year of Innovation in Plant-Based Drinks, Yogurts & Ice Cream, 2023.

[7] Rasane P., Jha A., Sabikhi L., Kumar A., Unnikrishnan V.S. (2015). Nutritional Advantages of Oats and Opportunities for Its Processing as Value Added Foods—A Review. J. Food Sci. Technol..

[8] Mäkinen O.E., Sozer N., Ercili-Cura D., Poutanen K. (2016). Protein From Oat: Structure, Processes, Functionality, and Nutrition. Sustainable Protein Sources.

[9] Collard K.M., McCormick D.P. (2021). A Nutritional Comparison of Cow’s Milk and Alternative Milk Products. Acad. Pediatr..

[10] Daou C., Zhang H. (2012). Oat Beta-Glucan: Its Role in Health Promotion and Prevention of Diseases. Compr. Rev. Food Sci. Food Saf..

[11] Skendi A., Biliaderis C.G., Lazaridou A., Izydorczyk M.S. (2003). Structure and Rheological Properties of Water Soluble β-Glucans from Oat Cultivars of Avena Sativa and Avena Bysantina. J. Cereal Sci..

[12] FDA U.S Food and Drug Administration. https://www.accessdata.fda.gov/scripts/cdrh/cfdocs/cfcfr/cfrsearch.cfm?fr=101.81.

[13] Health Canada. https://www.canada.ca/content/dam/hc-sc/migration/hc-sc/fn-an/alt_formats/pdf/label-etiquet/claims-reclam/assess-evalu/oat_avoine-eng.pdf.

[14] Huang K., Zhang S., Guan X., Li C., Li S., Liu Y., Shi J. (2021). Effect of the Oat β-Glucan on the Development of Functional Quinoa (Chenopodium Quinoa Wild) Milk. Food Chem..

[15] EFSA Panel on Dietetic Products, Nutrition and Allergies (NDA) (2011). Scientific Opinion on the Substantiation of Health Claims Related to Beta-Glucans from Oats and Barley and Maintenance of Normal Blood LDL-Cholesterol Concentrations (ID 1236, 1299), Increase in Satiety Leading to a Reduction in Energy Intake (ID 851, 852), Reduction of Post-Prandial Glycaemic Responses (ID 821, 824), and “Digestive Function” (ID 850) Pursuant to Article 13(1) of Regulation (EC) No 1924/2006. EFSA J..

[16] Biörklund M., Van Rees A., Mensink R.P., Onning G. (2005). Changes in Serum Lipids and Postprandial Glucose and Insulin Concentrations after Consumption of Beverages with B-Glucans from Oats or Barley: A Randomised Dose-Controlled Trial. Eur. J. Clin. Nutr..

[17] Srivastava N. (2020). Patent Insights: Beta Glucan as Functional Food Component.

[18] Srivastava N. (2023). Patent Insights: Nutrition and Gut Health for Immunity.

[19] Angelov A., Yaneva-Marinova T., Gotcheva V. (2018). Oats as a Matrix of Choice for Developing Fermented Functional Beverages. J. Food Sci. Technol..

[20] Vasquez-Orejarena E., Simons C.T., Litchfield J.H., Alvarez V.B. (2018). Functional Properties of a High Protein Beverage Stabilized with Oat-β-Glucan. J. Food Sci..

[21] Gorman M., Knowles S., Falkeisen A., Barker S., Moss R., McSweeney M.B. (2021). Consumer Perception of Milk and Plant-Based Alternatives Added to Coffee. Beverages.

[22] Pastre T. (2022). Beverage Market Outlook 2022: Inflation, Supply Chains, & Trends in Beverage Consumption.

[23] Zhou S., Jia Q., Cui L., Dai Y., Li R., Tang J., Lu J. (2023). Physical–Chemical and Sensory Quality of Oat Milk Produced Using Different Cultivars. Foods.

[24] (2007). Sensory Analysis—General Guidelines for the Design of Test Rooms.

[25] Wan W., Xu B. (2018). Development of an Orange Juice Beverage Formulated with Oat Beta-Glucan and Whey Protein Isolate. J. Sci. Food Agric..

[26] Oliveira D., Deliza R. (2021). Comparison of Consumer-Based Methodologies for Optimizing the Development of New Products: A Case Study with Probiotic Chocolate Flavored Milk. Food Sci. Technol. Int..

[27] Lawless H.T., Heymann H. (2010). Sensory Evaluation of Food: Principles and Practices.

[28] Neville M., Tarrega A., Hewson L., Foster T. (2017). Consumer-Orientated Development of Hybrid Beef Burger and Sausage Analogues. Food Sci. Nutr..

[29] Almli V.L., Næs T. (2018). Conjoint Analysis in Sensory and Consumer Science: Principles, Applications, and Future Perspectives. Methods in Consumer Research, Volume 1: New Approaches to Classic Methods.

[30] Moss R., Barker S., Falkeisen A., Gorman M., Knowles S., McSweeney M.B. (2022). An Investigation into Consumer Perception and Attitudes towards Plant-Based Alternatives to Milk. Food Res. Int..

[31] Ares G., Barreiro C., Deliza R., Giménez A., Gámbaro A. (2010). Application of a Check-All-That-Apply Question to the Development of Chocolate Milk Desserts. J. Sens. Stud..

[32] Multiple Factor Analysis (MFA) Statistical Software for Excel. https://www.xlstat.com/en/solutions/features/multiple-factor-analysis-mfa.

[33] Waxman H. (2017). Dairy and Dairy Alternative Beverages Trends in the U.S..

[34] Vaikma H., Kaleda A., Rosend J., Rosenvald S. (2021). Market Mapping of Plant-Based Milk Alternatives by Using Sensory (RATA) and GC Analysis. Future Foods.

[35] Sridhar K., Bouhallab S., Croguennec T., Renard D., Lechevalier V. (2022). Recent Trends in Design of Healthier Plant-Based Alternatives: Nutritional Profile, Gastrointestinal Digestion, and Consumer Perception. Crit. Rev. Food Sci. Nutr..

[36] International Food Information Council 2022 Food and Health Survey. https://foodinsight.org/2022-food-and-health-survey/.

[37] Hovardas T., Korfiatis K.J. (2006). Word Associations as a Tool for Assessing Conceptual Change in Science Education. Learn Instr..

[38] Ares G., Giménez A., Gámbaro A. (2008). Understanding Consumers’ Perception of Conventional and Functional Yogurts Using Word Association and Hard Laddering. Food Qual. Prefer..

[39] Roininen K., Arvola A., Lähteenmäki L. (2006). Exploring Consumers’ Perceptions of Local Food with Two Different Qualitative Techniques: Laddering and Word Association. Food Qual. Prefer..

[40] Erdem T., Swait J. (2004). Brand Credibility, Brand Consideration, and Choice. J. Consum. Res..

[41] Paries M., Bougeard S., Vigneau E. (2022). Multivariate Analysis of Just-About-Right Data with Optimal Scaling Approach. Food Qual. Prefer..

[42] Iserliyska D., Dzhivoderova M., Nikovska K. (2017). Application of penalty analysis to interpret jar data—A case study on orange juices. Curr. Trends Nat. Sci..

[43] Khorshidian N., Yousefi M., Shadnoush M., Mortazavian A.M. (2018). An Overview of β-Glucan Functionality in Dairy Products. Curr. Nutr. Food Sci..

[44] Yang Z., Xie C., Bao Y., Liu F., Wang H., Wang Y. (2023). Oat: Current State and Challenges in Plant-Based Food Applications. Trends Food Sci. Technol..

[45] McClements D.J. (2020). Development of Next-Generation Nutritionally Fortified Plant-Based Milk Substitutes: Structural Design Principles. Foods.

[46] Kumar L., Sehrawat R., Kong Y. (2021). Oat Proteins: A Perspective on Functional Properties. LWT.

[47] Matta Z., Chambers IV E., Garcia J.M., Helverson J.M.G. (2006). Sensory Characteristics of Beverages Prepared with Commercial Thickeners Used for Dysphagia Diets. J. Am. Diet Assoc..

[48] Paul A.A., Kumar S., Kumar V., Sharma R. (2019). Milk Analog: Plant Based Alternatives to Conventional Milk, Production, Potential and Health Concerns. Crit. Rev. Food Sci. Nut..

[49] Abdi H., Williams L.J., Valentin D. (2013). Multiple Factor Analysis: Principal Component Analysis for Multitable and Multiblock Data Sets. Wiley Interdiscip. Rev. Comput. Stat..

[50] Corvera-Paredes B., Sánchez-Reséndiz A.I., Medina D.I., Espiricueta-Candelaria R.S., Serna-Saldívar S., Chuck-Hernández C. (2022). Soft Tribology and Its Relationship With the Sensory Perception in Dairy Products: A Review. Front. Nutr..

[51] McGorrin R.J. (2019). Key Aroma Compounds in Oats and Oat Cereals. J. Agric. Food Chem..

[52] Ares G., Giménez A., Gámbaro A. (2009). Consumer Perceived Healthiness and Willingness to Try Functional Milk Desserts. Influence of Ingredient, Ingredient Name and Health Claim. Food Qual. Prefer..

[53] Baker M.T., Lu P., Parrella J.A., Leggette H.R. (2022). Consumer Acceptance toward Functional Foods: A Scoping Review. Int. J. Environ. Res. Public Health.

[54] Cardello A.V., Llobell F., Giacalone D., Roigard C.M., Jaeger S.R. (2022). Plant-Based Alternatives vs. Dairy Milk: Consumer Segments and Their Sensory, Emotional, Cognitive and Situational Use Responses to Tasted Products. Food Qual. Prefer..

